# Isolation and Characterization of Potassium-Solubilizing Rhizobacteria (KSR) Promoting Cotton Growth in Saline–Sodic Regions

**DOI:** 10.3390/microorganisms12071474

**Published:** 2024-07-19

**Authors:** Yue Zhao, Hongbang Liang, Jihong Zhang, Yu Chen, Yam Prasad Dhital, Tao Zhao, Zhenhua Wang

**Affiliations:** 1 College of Water Conservancy & Architectural Engineering, Shihezi University, Shihezi 832000, China; 20222010033@stu.shzu.edu.cn (Y.Z.); 20222410113@stu.shzu.edu.cn (H.L.); zhangjihong_eric@163.com (J.Z.); chenyu2@stu.shzu.edu.cn (Y.C.); ypdhital@gmail.com (Y.P.D.); 20232010043@stu.shzu.edu.cn (T.Z.); 2 Key Laboratory of Modern Water-Saving Irrigation of Xinjiang Production & Construction Group, Shihezi University, Shihezi 832000, China; 3 Technology Innovation Center for Agricultural Water & Fertilizer Efficiency Equipment of Xinjiang Production & Construction Group, Shihezi 832000, China; 4 Key Laboratory of Northwest Oasis Water-Saving Agriculture, Ministry of Agriculture and Rural Affairs, Shihezi 832000, China

**Keywords:** potassium-solubilizing rhizobacteria, cotton rhizosphere soil, potassium utilization efficiency, alkaline tolerance, organic acid, growth promotion

## Abstract

Cotton is highly sensitive to potassium, and Xinjiang, China’s leading cotton-producing region, faces a severe challenge due to reduced soil potassium availability. Biofertilizers, particularly potassium-solubilizing rhizobacteria (KSR), convert insoluble potassium into plant-usable forms, offering a sustainable solution for evergreen agriculture. This study isolated and characterized KSR from cotton, elucidated their potassium solubilization mechanisms, and evaluated the effects of inoculating KSR strains on cotton seedlings. Twenty-three KSR strains were isolated from cotton rhizosphere soil using modified Aleksandrov medium. Their solubilizing capacities were assessed in a liquid medium. Strain A10 exhibited the highest potassium solubilization capacity (21.8 ppm) by secreting organic acids such as lactic, citric, acetic, and succinic acid, lowering the pH and facilitating potassium release. A growth curve analysis and potassium solubilization tests of A10 under alkali stress showed its vigorous growth and maintained solubilization ability at pH 8–9, with significant inhibition at pH 10. Furthermore, 16S rRNA sequencing identified strain A10 as *Pseudomonas aeruginosa*. Greenhouse pot experiments showed that inoculating cotton plants with strain A10 significantly increased plant height and promoted root growth. This inoculation also enhanced dry biomass accumulation in both the aerial parts and root systems of the plants, while reducing the root–shoot ratio. These results suggest that *Pseudomonas aeruginosa* A10 has potential as a biofertilizer, offering a new strategy for sustainable agriculture.

## 1. Introduction

Potassium, an essential macronutrient, is crucial for crop growth and stress responses [1]. Cotton is particularly susceptible to potassium deficiency due to its sparsely distributed root architecture [2]. Potassium deficiency during the growth stages of cotton can inhibit root and leaf respiration and photosynthesis, restrict root elongation, and consequently impair the plant’s nutrient absorption. This deficiency may lead to reduced plant height, leaf area, and overall biomass [3]. The physiological characteristics of cotton make it sensitive to potassium, often resulting in a mild deficiency even in potassium-sufficient soils [4]. Enhancing the soil’s potassium content can effectively increase the biomass of functional leaves, improve carbon metabolism in the leaves, and raise the seeds’ cotton yield [5]. Therefore, developing effective strategies for optimizing soil potassium utilization is imperative for ensuring the healthy growth and productivity of cotton.

The Xinjiang cotton region is a pivotal hub within the global cotton industry, generating 80% of China’s and 20% of the world’s cotton output. Moreover, there is additional potential for yield enhancement, which is estimated to be as high as 71% [6]. Recent investigations have revealed a pronounced depletion of total soil potassium in this region [7], accompanied by a concurrent decline in readily available potassium levels [8]. These factors are deemed critical in influencing both soil fertility and cotton yield [9]. China, the foremost consumer of chemical potash fertilizers worldwide, heavily relies on imports, with importation rates reaching as high as 50% [10]. However, the widespread use of chemical fertilizers is associated with groundwater contamination, reductions in soil organic matter, the destabilization of soil architecture, and greenhouse gas emissions [11,12,13,14]. In addition, the cotton industry in Xinjiang faces challenges due to soil salinization [15]. Generally, its soil is alkaline, with a pH maintained above 8.3 [16]. In response to the increasing potassium demand within the Xinjiang cotton industry and the pressing need to alleviate environmental stress, developing strategies to reduce the reliance on inorganic potassium fertilizers is imperative.

Potassium-solubilizing rhizobacteria (KSR) exhibit considerable promise in agriculture as eco-friendly, nature-based solutions for mitigating the rhizospheric potassium dilemma [17,18]. These bacteria use biochemical processes such as acidolysis, complexation, and chelation to convert fixed potassium into plant-available forms [19]. These mechanisms reduce reliance on chemical potassium fertilizers while promoting environmental sustainability. Globally, the focus on KSR’s research and application has intensified, leading to the identification of various effective strains. For example, scientists have cultured KSR strains from the soils of monsoon crops like corn and banana, such as *Rhizobium pusense*, *Rhizobium rosettiformans*, *Flavobacterium anhuiense clade*, and *Agrobacterium tumefaciens* [11]. Similarly, the efficient KSR strains *Streptomyces sundarbansensis*, *Burkholderia genus*, and *Streptomyces rochei* have been isolated from the rhizospheric soil of *Mikania micrantha* [20,21]. Additionally, under cultivation with insoluble potassium sources, the *Priestia aryabhattay* strain SK1-7 was observed to secrete elevated levels of organic acids and polysaccharides, increasing the activation of genes associated with potassium solubilizing, thereby facilitating potassium solubilization [22]. Consequently, KSR serve as a natural method to boost soil potassium availability, providing a feasible approach to decreasing our dependence on chemical fertilizers and supporting sustainable agricultural practices worldwide.

In practical applications, KSR have showcased their substantial efficacy. Studies have confirmed their capability to significantly enhance soil potassium availability, ultimately improving crop yields. For instance, three KSR strains, *Pseudomonas orientalis*, *Rahnella aquatilis*, and *Pantoea agglomerans*, were isolated from rice rhizosphere soil and inoculated into the soil, resulting in increased rice grain yield and potassium utilization efficiency [23]. Moreover, inoculating potting soil with *Pseudomonas sp.* S19-1 and adding muscovite improved the potassium absorption of tomato roots, leading to a 12.5% increase in the yield of the potted plants compared to the control [24]. In recent years, KSR have been validated as an effective remedy for enhancing plant growth in response to both biotic and abiotic stressors [17]. For instance, under salt stress, strains including *Pantoea agglomerans*, *Pseudomonas putida*, *Citrobacter braakii*, *Erwinia iniecta*, and *Enterobacter hormaechei* have significantly elevated the cytoplasmic K^+^/Na^+^ ratio in rice, thereby fostering rice growth and yield improvement [25]. Additionally, researchers believe that native microorganisms, rather than exotic species, are more likely to be recruited by crop roots and occupy a stable niche, providing a safer and more sustainable method for developing microbial products [26]. Furthermore, the intrinsic growth and metabolic functions of KSR significantly contribute to the biogeochemical cycling of potassium within the soil matrix. Potassium is involved in vital biological activities, such as maintaining the osmotic potential of bacteria, radicalizing various intracellular enzymes, and regulating intracellular pH [27]. A study [28] conducted in Ohio, USA, underscored the significance of soil microbial potassium as a substantial potassium reservoir that warrants attention. Consequently, KSR have emerged as a promising strategy for rhizospheric potassium management, facilitating efficient potassium utilization and bolstering crop productivity.

Although various functional soil microorganisms have been found in jujube rhizospheric soil in Xinjiang [29], research on cotton rhizospheric microorganisms, particularly KSR, is limited. Given the potassium demand of cotton and the growth pressure it experiences under alkali conditions, the objective of this study is to isolate KSR from the cotton fields in Xinjiang. Additionally, we aim to explore their ability to solubilize insoluble potassium minerals in liquid culture and the potential of microbial biomass potassium as an available alternative. Furthermore, we will assess the survival ability of KSR under environmental stress, conduct a molecular identification of efficient KSR, and further investigate their potassium-solubilizing mechanisms and effects on cotton seedlings’ growth. This research will provide technical support and a scientific basis for reducing chemical fertilizer usage, mitigating environmental burden, and enriching the potassium-solubilizing microbial resource library, thus promoting the sustainable development of agriculture.

## 2. Materials and Methods

### 2.1. Site Description

The site is located in the Xiayedi irrigated area, Xinjiang, China (85°33′–85°35′ E, 44°48′–44°50′ N), a typical area affected by salinization in China [16,30]. This region features a typical temperate continental climate and a loamy clay soil texture [15]. Since the late 1990s, local farmers have primarily cultivated cotton using mulched drip irrigation techniques and precise planting patterns to ensure optimal growth [31].

### 2.2. Soil Sampling

Soil samples were collected from the rhizosphere of cotton plants during their flowering stage in the Xiayedi irrigated area. A five-point sampling method was employed across three plots, each spaced over 200 m apart. At each point, four healthy cotton plants were selected. Their surface soil was removed, and roots with closely adhered soil (approximately 1 mm thick) were collected in aseptic bags. These samples were then transported to the laboratory under refrigeration. The roots were carefully transferred into sterile centrifuge tubes filled with 20 mL of 10 mM PBS solution, with 7.605 g NaCl, 0.9937 g Na_2_HPO_4_, and 0.468 g NaH_2_PO_4_ per L, and then vortexed at 120 r min^−1^ for 10 min. After removing the roots with sterile tweezers, the rhizospheric soil solution was stored in an ultralow-temperature freezer for further analysis.

### 2.3. Soil Properties Analysis

To determine soil properties, 20 g of soil samples were oven-dried at 105 °C for 24 h to measure their moisture content gravimetrically. Soil pH was determined using a 1:2.5 soil-to-water suspension. The total potassium was extracted using a 7:1 extraction ratio of a mixture of HNO_3_ and HCIO_4_, which was maintained for 3 h. Then, the mixture was diluted to 100 mL and analyzed using flame atomic absorption spectrometry. Available potassium was measured by atomic absorption spectrometry after its extraction with 3% ammonium carbonate. Total nitrogen and phosphorus were quantified through the Kjeldahl and the molybdenum blue method [21].

### 2.4. Mineral Samples and Medium

Potassium feldspar powder, manufactured by Guzhang County Shanlin Stone Language Mine Products Co., Ltd., Xiangxi Autonomous Prefecture, China, was sieved through a 0.15 mm aperture, soaked in sterilized water, and left for 72 h to remove soluble potassium. A modified solid Aleksandrov medium (MAM_s_), containing 5.0 g of glucose, 0.3 g of magnesium sulfate, 2.0 g of disodium hydrogen phosphate, 0.03 g of ferrous sulfate, 0.03 g of manganese sulfate, and 2.0 g of potassium feldspar per L, was solidified with 15.0 g of agar and included 0.25 g of bromothymol blue as a chromogenic reagent, or was diluted to 1 L with deionized water. The Lysogeny broth (LB) medium contained 10 g of tryptone, 5 g of yeast extract, and 10 g of NaCl per L, solidified with 20 g of agar or brought to 1 L using deionized water.

### 2.5. Isolation of KSR from Cotton Rhizosphere

Rhizospheric soil samples were obtained by subjecting the soil–root solution to low-temperature centrifugation. Under sterile conditions, 10 g of the sample was added to a conical flask containing glass beads and 90 mL of sterile water. This mixture was shaken at 150 r min^−1^ and 30 °C for 30 min, and settled for 20 min after that. The soil samples were serially diluted in physiological saline to a population of 10^−5^ shaking mL^−1^. A 50 µL aliquot from each dilution ranging between 10^−4^ and 10^−5^ was plated on LB medium and spread evenly. The plates were maintained at 28 ± 1°. Colonies with distinct morphologies were selected for purification, while those with similar morphologies were grouped, labeled, and inoculated on slant media for storage at 4 °C. KSR isolated from the rhizosphere of cotton were further screened using a modified solid Aleksandrov medium containing potassium feldspar as the sole potassium source. Each strain was inoculated four times on Aleksandrov solid medium and incubated at 28 ± 1 °C for 20, 36, and 48 h. The diameters of the potassium solubilization zones (D) and the colonies (d) were measured to calculate their solubilization efficiency (KE), where KE = D/d [23].

### 2.6. Potassium Solubilizing Ability of KSR

The KSR strains were cultured in LB medium and shaken overnight (30 °C, 150 r min^−1^, OD_600_ 0.5) to increase the population to 10^8^ CFU mL^−1^. A 50 mL aliquot of Aleksandrov solution was introduced into a 150 mL flask, which was then sterilized at 121 °C under pressure for 30 min. After the flask had cooled, 1 mL of the KSR solution was inoculated into it. The flasks were incubated with shaking at 150 r min^−1^ and at 28 ± 1 °C for 7 days, with three replicates per treatment. Disposable sampling was conducted on days 1, 3, and 7. The concentration of extracellular soluble potassium (ESK) was measured utilizing a flame photometer. Additionally, 2 mL of the bacterial fermentation liquid was subjected to three freeze/thaw cycles (−20 °C/28 °C) to lyse the cells. Then, 1 mL of 6% H_2_O_2_ was added to the mixture and incubated in a 100 °C water bath for 1 h to dissolve extracellular polymeric substances (EPSs). Sterile deionized water was added to restore the mixture to its original volume, followed by centrifugation at 5000 r min^−1^ for 10 min. The concentration of total soluble potassium (TSK) in the supernatant was determined by a flame photometer. The ESK and TSK were measured to calculate the intracellular soluble potassium (ISK), where ISK = TSK − ESK [27].

The pH of the Aleksandrov solution was adjusted to 8, 9, and 10 using a NaOH solution. The KSR strains were inoculated into the broth, with three replicates for each strain. After 24 h of incubation at a constant temperature, the TSK concentration was measured. The specific procedures followed were the same as those previously described.

### 2.7. Assessment of Strain Viability Under Different pH Conditions

After preparation, the LB was adjusted to pH levels of 7, 8, 9, and 10 using a NaOH solution and then sterilized at 121 °C. To sterilize each well of a 24-well clear-bottom microplate, 2 mL of anhydrous ethanol was added, followed by a 5 min standing period. After the ethanol was discarded, the microplate was exposed to 254 nm ultraviolet light for 15 min. To prevent condensation on the lid during measurements, 2 mL of 0.05% Triton X-100 solution (containing 20% ethanol) was applied to the lid and left for 15 s, and then removed. The microplate was air-dried before measurements. The strains were cultured for 12 h, followed by centrifugation at 1000 r min^−1^ for 5 min. The supernatant was then removed. The bacterial pellet was resuspended in distilled water and gently stirred to ensure even distribution. The suspension was diluted to an approximate optical density (OD600) of 0.5. Subsequently, 0.5 mL of this bacterium liquid was injected into a 24-well plate containing 1.5 mL of broth adjusted to various pH levels. During inoculation, the suspension was slowly injected and gently swirled to distribute the bacteria evenly. The first well was not inoculated and was filled with an equal volume of culture medium, serving as a control to verify whether the microplate was contaminated. The microplate was then covered with the pretreated lid. The 24-well plate was placed in a microplate reader (BioTek Synergy LX, Winooski, Vermont, USA) at 37 °C, with shaking at 355 rpm to prevent the bacteria from settling at the bottom of the wells. Every 160 s, the shaking was paused to measure the absorbance, and this process continued for 24 h. The absorbance measurements served as an indicator of KSR viability under different pH conditions, with elevated absorbance values indicating enhanced bacterial growth in the new environment.

### 2.8. DNA Sequencing and Phylogenic Analysis of KSR

The KSR’s morphological attributes were evaluated through the examination of colony morphology, the presence or absence of spores, and Gram staining reactions. The isolated bacteria were molecularly identified through the utilization of the colony polymerase chain reaction (PCR) technique [24]. A PCR amplification system was established using the universal bacterial primers F27 (5′-AGAGTTTGATCCTGGCTCAG-3′) and R1492 (5′-GGTTACCTTGTTACGACTT-3′). The reaction mixture for the PCR contained 1.0 µL of genomic DNA (20 ng µL^−1^), 5.0 µL of 10X Buffer (containing 2.5 mM Mg^2+^), 1.0 µL of Taq polymerase (5 U µL^−1^), 1.0 µL of dNTPs (10 mM), 1.5 µL of primer 27F (10 µM), 1.5 µL of primer 1492R (10 µM), and 39.0 µL of ddH2O, totaling 50 µL. The PCR amplification protocol involved an initial denaturation at 95 °C for 5 min; followed by 35 cycles of 95 °C for 30 s, 58 °C for 30 s, and 72 °C for 1 min 30 s; and a final extension at 72 °C for 7 min. Following the reaction, 3 µL of the PCR product was subjected to 1% agarose gel electrophoresis to verify the amplified fragments. The PCR products were then purified using the AxyPrep DNA Gel Recovery Kit (Axygen Biosciences, Union City, CA, USA). The purified PCR products from each strain were sequenced using the ABI3730-XL sequencer. Sequence alignment was performed using the NCBI Blast tool to compare the sequences with the NCBI 16S database, identifying the species with the highest sequence similarity to the test specimens. Phylogenetic trees were constructed using MEGA 11 software.

### 2.9. Determination of Organic Acids and Enzyme Types and Contents

The KSR strains were cultured in broth to increase the population to 10^8^ CFU mL^−1^. Then, 1 mL of the culture was inoculated into 50 mL of fermentation medium containing an insoluble potassium source (potassium feldspar), with three replicates per group. After incubation (30 °C, 7 days, 150 r min^−1^) and subsequent centrifugation (4 °C, 10 min, 12,000 r min^−1^), the supernatant of the 1 mL cultures was collected. After filtration through a syringe filter, the samples were ready for further analysis. High-performance liquid chromatography (HPLC) was used to determine the varieties and content of their organic acids. The chromatographic parameters were set as follows: Shimadzu LC-20AT (Shimadzu Corporation Kyoto, Japan), UV detector SPD-20A, column oven CTO-20AC, C18 reversed-phase column (150 mm × 4.6 mm, 5 μm), mobile phase A: 100% methanol, B: 0.2% aqueous solution of sodium dihydrogen phosphate (pH 2.7) (A: B = 3:97), injection volume 10 μL, flow rate 0.6 mL min^−1^, column temperature 30 °C, and UV wavelength 210 nm.

In addition, 5 mL of supernatant was collected from the fermentation broth, and its enzymatic activity was determined using a double antibody sandwich method and spectrophotometry. Taking β-glucuronidase (GUS) as an example, 50 μL of purified GUS antibody was added to coat the wells of a microplate, forming a solid-phase antibody. Subsequently, 50 μL of the broth was sequentially injected into the wells and maintained at 37 °C for 30 min. After removing the liquid, the plate was tapped dry, and a washing solution was added into wells. After standing for 30 s, the solution was discarded. The plate was washed five times. Then, the plate was incubated with HRP-labeled GUS antibody (Jingmei Biotechnology, Yancheng, China) at 37 °C for another 30 min to form the antibody–antigen-enzyme-labeled antibody complex. The plate was washed five times again. After adding 100 μL of TMB substrate (3,3′,5,5′-tetramethylbenzidine) into the wells, these samples were incubated in the dark at 37 °C for 10 min. TMB was initially converted into blue by HRP enzyme catalysis and then into yellow under acidic conditions. The color intensity showed a positive correlation with the GUS content. The absorbance at 450 nm was recorded with a microplate reader, and the GUS concentration was calculated utilizing a standard curve.

Moreover, the pH of the liquid medium was adjusted to 8, 9, and 10, respectively, and strain A10 was inoculated. Each pH level was tested in triplicate. After 24 h of culture, the varieties and concentrations of organic acids present were measured. The specific procedures followed were the same as those previously described.

### 2.10. Pot Experiment

The pot experiment, conducted within the controlled environment of the artificial climate chamber at Shihezi University, included four treatments: control (sterile solution), K (potassium feldspar with sterile solution), A10 (bacteria solution), and K + A10 (potassium feldspar with bacteria solution). The soil was pretreated before the pot experiment. The soil, collected from cotton fields in Xinjiang’s Xiayedi irrigated area, was sterilized twice at 121 °C for 15 min each time. The pH of this soil sample was 8.28. Each pot contained 11 kg of soil and was supplemented with 0.36 g of urea (N: 46%) and 0.17 g of diammonium phosphate (N: 18%, P_2_O_5_: 46%). For the K and K + A10 treatments, 0.4 g of potassium feldspar powder was added to the soil and mixed thoroughly. Each treatment was replicated five times. Approximately 1 L of water was poured into each pot, which then stood for 24 h. After 24 h, two-week-old cotton seedlings with uniform growth were selected from the nursery, and three seedlings were transplanted into each pot. The climate chamber was maintained on a 12 h day/night cycle (28 °C/22 °C) with a relative humidity of 65%. The KSR strains were cultured in broth to increase their population to 10^8^ CFU mL^−1^ and then diluted tenfold with distilled water. One week after transplantation, 50 mL of the diluted bacterial solution was inoculated on the roots of cotton seedlings in the A10 and K + A10 treatments. The control and K treatments received an equal amount of diluted sterile solution. The inoculation was performed again after one week. Seven days later, the soil and plants in the pots were completely excavated. Their roots were washed with running water to ensure they were completely retained. Plant height was measured, and total root length was determined using a root scanner. The seedlings were dried to a constant weight to measure their dry weight. The root–shoot ratio was determined by the division of root dry weight by the shoot dry weight.

### 2.11. Data Analysis

Experimental data were analyzed using IBM SPSS 26.0 (SPSS Inc., Chicago, IL, USA). The amount of potassium released by the KSR strains under alkaline conditions; the organic acid content and enzyme activity of the KSR A10 pure culture; and the plant height, total root length, and dry weight of the cotton seedlings were analyzed using a one-way analysis of variance (ANOVA) and a least significant difference (LSD) test at a 95% confidence level (*p* < 0.05). Before the analysis of variance, all aggregated data were tested for the homogeneity of their variance. Figures were plotted with Origin Pro 2023 Education Version (OriginLab, Northampton, MA, USA) and RStudio version 4.3.2 (2023.09.1 + 494).

## 3. Results

### 3.1. Isolation and Potassium Solubilizing Ativity of Cotton Rhizosphere KSR

**Figure 1:**
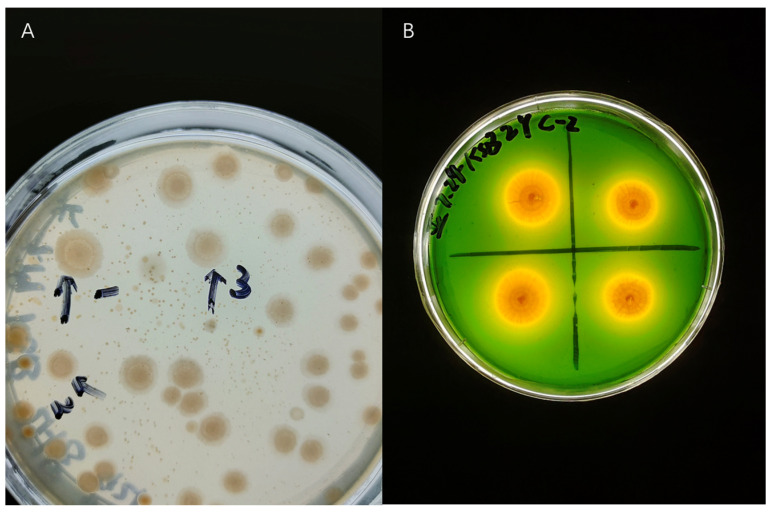
Samples of KSR isolates from the (**A**) LB agar dilution phase and (**B**) the MAMs purification culture phase.

**Figure 2:**
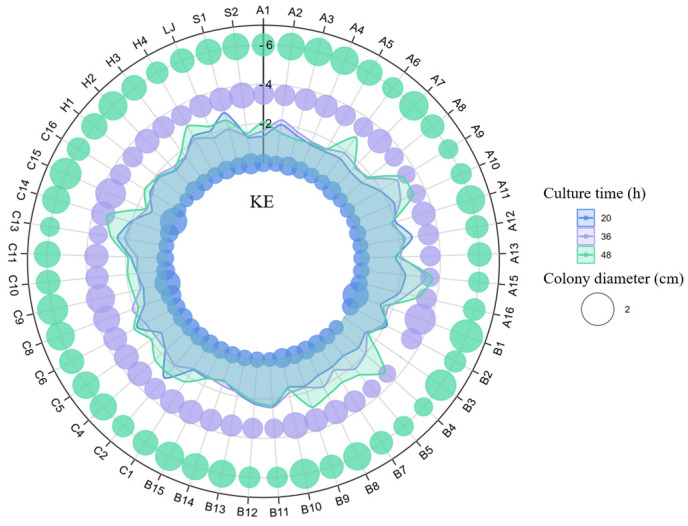
Phosphate-solubilizing ability of KBS on agar medium. Each value is the average of a minimum of four replicates.

To identify and develop efficient KSR, we isolated 77 strains of various colony types from cotton rhizosphere soil in Xinjiang (Figure 1). The soil had a pH of approximately 8.28 and contained 148.11 mg·kg^−1^ of available potassium (Table 1). Forty-nine of the screened isolates demonstrated the ability to increase potassium solubility, with solubilization indices (D/d) ranging from 1.16 to 3.71 (Figure 2). Potassium solubilization dynamics varied among strains; for instance, strain A2 peaked at 36 h before declining, whereas strains such as B7 showed a gradual increase over time. Strain A15 had the highest solubilization capability on agar plates.

**Figure 3:**
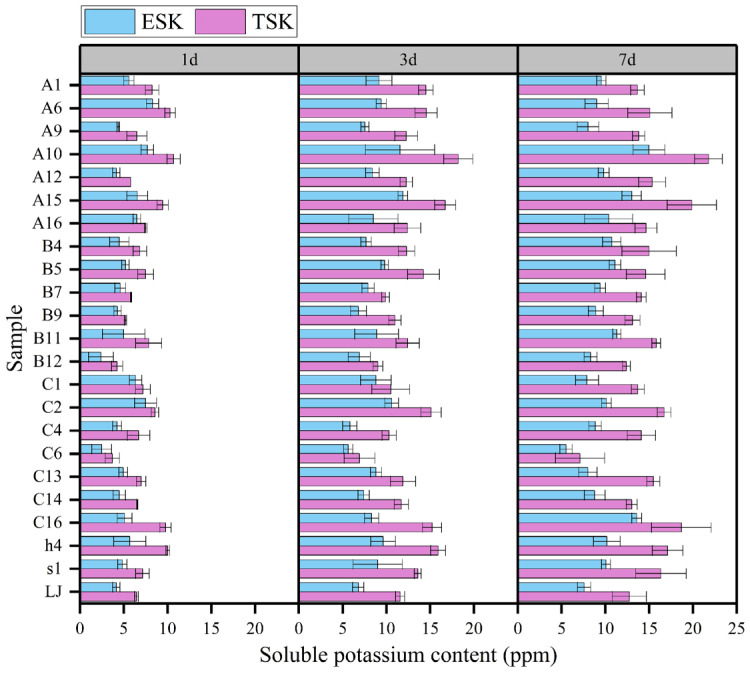
The potassium-solubilizing activity of different KSR in broth at different days of inoculation. Each data point represents the mean of at least three replicates. Error bars represent standard deviations. The X-axis range of each panel is 25 ppm.

Based on their potassium efficiency (KE) values, twenty-three strains were selected, in descending order: B4, A10, C14, A9, A16, A6, H4, B9, C13, B5, C2, C4, C6, S1, LJ, B11, A12, B7, C1, B12, A1, and C16. These strains were inoculated in liquid media to quantitatively measure their potassium solubilization capabilities. The results indicated that strains A6, C1, C2, C6, and C13 led to a decrease in the soluble potassium concentration by 0.13 to 0.9 ppm on the seventh day, compared to the third day, without H_2_O_2_ treatment. In contrast, the concentration of soluble potassium in the broth of other KSR strains significantly increased over time (Figure 3). Specifically, the TSK values of KSR ranged from 3.70 to 10.7 ppm, 6.93 to 18.20 ppm, and 7.1 to 21.78 ppm on days 1, 3, and 7, respectively. The ESK and ISK values followed a similar trend. The ESK ranged from 2.40 to 8.30 ppm, 5.65 to 11.86 ppm, and 5.5 to 14.98 ppm on days 1, 3, and 7, respectively. The ISK ranged from 0.85 to 4.68 ppm, 1.30 to 6.90 ppm, and 1.60 to 7.47 ppm on days 1, 3, and 7, respectively. In terms of temporal scale, the soluble potassium concentration within the fermentation broth of each strain exhibited rapid growth from the first to the third day, followed by a slower rate of increase from the third to the seventh day, with increments ranging from 3.3 to 7.5 ppm and 0.17 to 4.2 ppm, respectively. Strain A10 was identified as having the highest capability for potassium release, achieving a concentration of 21.78 ppm after 7 days. This was followed by strains A15 and C16, which reached 19.87 ppm and 18.67 ppm, respectively. In contrast, strain C6 displayed the lowest potassium solubilization, with a release of 7.1 ppm.

**Figure 4:**
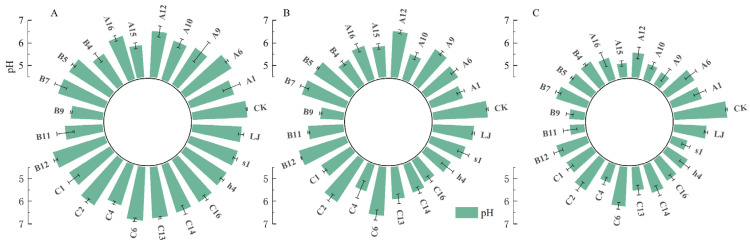
The impact of KSR on broth pH over various inoculation periods; (**A**–**C**) represent 1, 3, and 7 days, respectively. The initial pH of the broth was 7.0. Each data point represents the mean of at least three replicates. Error bars represent standard deviations.

**Figure 5:**
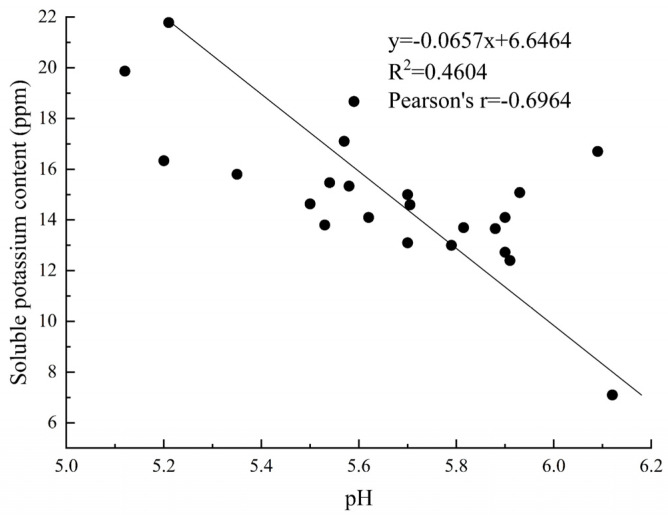
Correlation between potassium solubilizing capacity and pH value of culture medium.

We measured the pH values of 23 KSR strains over a 7-day incubation period (Figure 4). Compared to the potassium feldspar medium without inoculation, the KSR strains reduced the broth’s pH to a range of 5.12 to 6.93. After 7 days, strain A15 exhibited the lowest pH among all the KSR strains, followed by A9, B11, and B9. The pH dynamics varied over time among the strains. For example, strains A15 and B9 saw a pH reduction to 5.92 and 5.91, respectively, after one day, which further decreased to 5.12 and 5.17 by day seven. Strains A9, A12, B11, and S1 maintained a pH above 6 until the third day but decreased to lower levels, between 5.13 and 5.58, by the seventh day. A Pearson correlation analysis revealed a highly significant negative correlation between potassium solubilization and culture medium pH (*p* < 0.01, R^2^ = 0.4604, *r* = −0.6964) (Figure 5), indicating that lower pH values were associated with higher potassium solubilization.

### 3.2. Effect of Alkaline Stress on KSR

**Figure 6:**
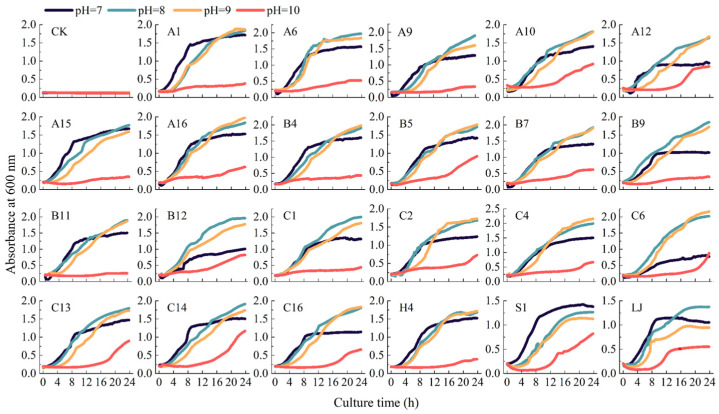
Growth curve of KSR under varying alkalinity.

The growth curves of KSR under alkaline stress revealed their adaptability to weak alkaline environments at pH values of 7, 8, and 9. However, at pH 10, their growth was significantly inhibited, as KSR experienced prolonged lag phases, shortened or attenuated exponential phases, and an absence or reduction in stationary phases (Figure 6).

Specifically, the KSR exhibited slow growth rates within 2 h, indicating a lag phase, under pH 7 conditions. However, at pH 8 and 9, except for strains A10, B9, B12, and C6, the intensified alkaline stress reduced bacterial proliferation rates, resulting in shallower slopes of the growth curves and an extension of the lag phase to 4 h. After the lag phase, the KSR transitioned into their exponential phase. Except for strains A6, A16, B5, B7, and C1, which exhibited no obvious differences in the slopes of their growth curves, the slopes of all other KSR became less steep under alkaline stress conditions. Under pH 7, all strains entered the stationary phase after 8 h, whereas under pH 8 and 9 bacteria entered their stationary phase after 12–16 h, with higher absorbance values compared to those under pH 7. Strains A12, B9, C1, C14, S1, and LJ entered their death phase after 16–20 h under pH 7, while strains H4, S1, and LJ entered their death phase after 20 h under pH 8 and 9.

Overall, the potassium release by KSB was highest at pH 7 to 9, while it significantly decreased at pH 10. Strains A9, A10, A15, B4, B11, B12, C6, C13, C14, and H4 showed no significant difference in their potassium release across pH levels 7, 8, and 9, indicating their strong adaptability to alkaline conditions. Strain A10 exhibited a significantly higher ability to solubilize potassium at pH 7 to 10 compared to other strains. Furthermore, strains B11, B12, C4, C6, C13, C14, and LJ showed the highest potassium solubilization at pH 8. Other strains exhibited a trend of decreasing potassium solubilization with increasing alkaline stress (Table 2).

### 3.3. Molecular and Biochemical Identification of Efficient KSR Strains

**Figure 7:**
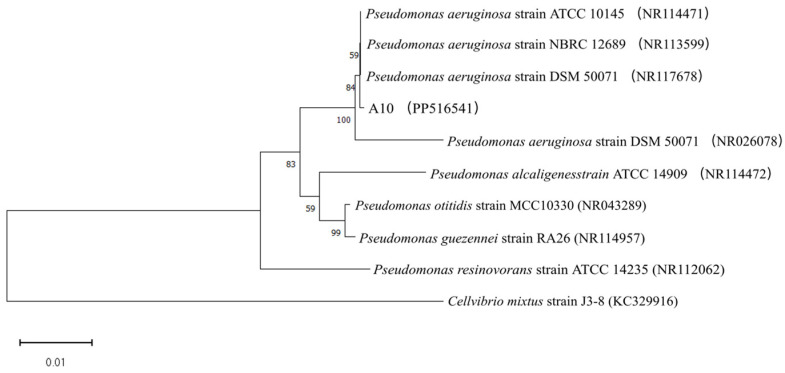
Phylogenetic tree of *Pseudomonas aeruginosa* A10 (PP516541) based on 16S rRNA sequences.

Based on the aforementioned research findings, strain A10 was selected as an efficient KSR strain for further identification. Following 48 h of cultivation at 28 °C on LB agar plates, strain A10 exhibited irregular colony edges, a pale yellow color, a flat morphology, smooth and moist surfaces, glossiness, an absence of mycelium, and adhesive properties, with a viscous texture (Table S1). The homology analysis of strain A10’s gene sequence was conducted using 16S rDNA, followed by the construction of a phylogenetic tree through the utilization of MEGA 11 (Figure 7), identifying it as *Pseudomonas aeruginosa*.

### 3.4. Quantification of Organic Acids and Enzymes

**Figure 8:**
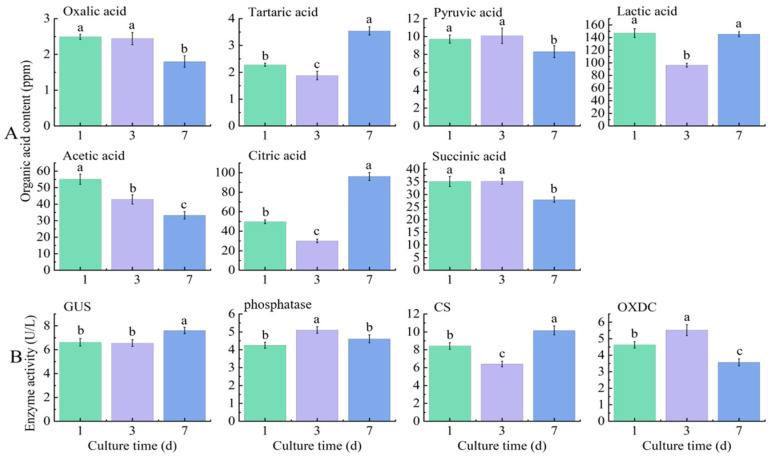
The organic acid content (**A**) and enzyme activity (**B**) of a pure KSR A10 culture at different days of inoculation. Each data point represents the mean of at least three replicates. Error bars represent standard deviations. The bars labeled with different letters indicate significant differences.

Under insoluble potassium source cultivation, strain A10 exhibited the highest lactate production: 147.08, 96.24, and 145.25 ppm on days 1, 3, and 7, respectively. Citrate, acetate, and succinate followed, with peak productions at 96.09, 55.12, and 35.25 ppm within 7 days, respectively. In contrast, oxalate, tartaric acid, and pyruvate showed lower yields, reaching maximum levels of 2.49, 3.54, and 10.08 ppm within 7 days. Additionally, strain A10 secreted β-glucuronidase (GUS), phosphatase, citrate synthase (CS), and oxalate decarboxylase (OXDC), with peak activities of 7.62, 5.10, 10.16, and 5.52 U/L, respectively, within 7 days. The dynamics of different organic acids and enzymes varied over time. Tartaric acid and citrate showed an initial decrease followed by an increase, while oxalic acid, pyruvate, lactate, acetate, and succinate showed an overall decreasing trend. The changes in enzyme activity were more complex, with citrate synthase and oxalate decarboxylase showing trends of an initial decline followed by an increase, and initial increase followed by a decline, respectively, consistent with the trends observed for citrate and oxalic acid (Figure 8).

Under varying pH conditions, A10 predominantly secreted lactic, acetic, citric, and succinic acid. The maximum concentrations of these acids were 147.09, 109.22, 107.36, and 57.00 μg/mL, respectively. In contrast, its secretion levels of oxalic, tartaric, and pyruvic acid remained relatively low, each below 10.00 μg/mL. The secretion patterns of these organic acids varied with pH, except for oxalic acid, which showed no significant change. As alkaline stress increased, the secretion of tartaric, pyruvic, and lactic acid decreased. Acetic acid secretion was minimal at pH 8 but increased with further alkaline stress. Conversely, the highest secretion levels of citric and succinic acid occurred at pH 8, with a subsequent decline as the alkalinity increased further (Table 3).

### 3.5. Promotional Effects of KSR on Cotton Growth

Finally, *Pseudomonas aeruginosa* A10 was tested in a pot experiment (Figure S1). Overall, compared to the control, the K + A10 treatment increased the total root length, root weight, plant height, and aboveground dry weight by 41, 23, 35, and 46%, respectively. Compared to the individual K or A10 treatments, the K + A10 treatment increased the total root length, root weight, plant height, and aboveground dry weight by 22, 16, 21, and 34%, and by 13, 10, 10, and 24%, respectively. Additionally, our study showed that the root–shoot ratio of cotton under the K, A10, and K + A10 treatments decreased from 52% to 51, 50, and 44%, respectively, compared to the control (Table 4).

## 4. Discussion

### 4.1. Isolation, Identification, and Evaluation of Potassium Solubilization Capacity of KSR

Plant-growth-promoting rhizobacteria are widely recognized as a nature-based solution for sustainable agriculture [11]. Our findings indicate that *Pseudomonas aeruginosa* A10 possesses a high capacity for potassium solubilization. This aligns with previous research that has recognized the genus *Pseudomonas* for its exceptional potassium-solubilizing capabilities. For instance, *Pseudomonas* strain S14-3 increased potassium release by 73% compared to controls [32]. Additionally, these bacteria can utilize root exudates and soil nutrients to gain a competitive advantage and suppress the proliferation of pathogens [33]. Furthermore, our research on ISK suggests that, during KSR growth, some potassium is either absorbed by the bacteria or adsorbed onto the viscous EPS they produce. After cell mineralization, this microbial biomass potassium is available for plant growth, thus serving as both a source and a reservoir of soil potassium [34].

In this study, isolated KSR strains exhibited varying potassium solubilization dynamics and capacities, likely associated with their secretion of extracellular secretory products (ESPs) and motility. During their growth and proliferation, bacteria continuously secrete various ESPs, including organic acids, inorganic acids, polysaccharides, and proteins. These ESPs facilitate the dissolution of insoluble potassium through mechanisms such as acidolysis, complexation, and chelation [17]. Polysaccharides and proteins are primary components of EPSs, which aggregate to form a complex three-dimensional network. This network provides structural support and a protective layer to the biofilm, creating a more competitive survival environment for the bacteria [35,36]. Moreover, microscopically, individual cells of KSR reach mineral surfaces through autonomous diffusion or passive transport (such as water shear flow or gravitational settling), growing and proliferating and exerting a potassium-solubilizing effect. The diffusion coefficient of mobile bacteria in aqueous environments is about 4 × 10^−10^ m^2^·s^−1^, while non-motile bacteria have a much lower coefficient of around 3 × 10^−13^ m^2^·s^−1^ [37], reflecting the advantageous environmental adaptability and resource acquisition of motile bacteria. In this study, KSR strains exhibited varying potassium solubilization dynamics and capacities, potentially influenced by the diversity of their ESPs and their motility.

### 4.2. The pH Dynamics and Mechanisms of Potassium Solution

The dynamic regulation of pH by KSR is critical to their growth and metabolic activity [19,27]. Our study showed a significant decrease in pH over time in the broth, underscoring the strong negative correlation between pH and the solubilization of potassium. This suggests that KSR acidifies the medium, likely through the production of various organic acids, a potential mechanism [19,38]. Therefore, we determined the types and concentrations of organic acids secreted by KSR. We found that KSR strain A10 primarily lowers environmental pH by secreting organic acids such as succinic, citric, acetic, and lactic acids, creating acidic conditions on the surface of potassium-rich minerals and facilitating their decomposition. This discovery aligns with numerous studies, which have documented that organic and inorganic acids secreted by KSR can actively enhance mineral dissolution by reducing the ambient pH, leading to gradual potassium ion mobilization and increased potassium availability [22,39,40]. Acidolysis, particularly corrosion by organic acids, is considered a primary mechanism of potassium’s solubilization by KSR [17,39]. KSR releases potassium ions from their crystal lattices by secreting various organic acids, such as lactic, tartaric, malic, oxalic, succinic, acetic, and citric acid [32,41]. Additionally, certain KSR strains, such as *Bacillus mucilaginosus*, can produce extracellular polysaccharides which exhibit a strong capacity to adsorb organic acids [42]. This ability facilitates their binding to mineral surfaces, creating high-concentration regions of organic acids near the minerals [38]. This phenomenon may explain the different pH reduction dynamics observed among various bacterial strains in our study. Moreover, citrate synthase facilitates citric acid synthesis, while oxalate decarboxylase is responsible for breaking down oxalic acid [43,44]. Their secretion levels corresponded to the variations in citric acid and oxalic acid over the cultivation period in our investigation. Interestingly, we also identified phosphatase secretion, suggesting a potential phosphorus-solubilizing capability of KSR strain A10 [45].

### 4.3. Survival Capability of KSR Under Alkaline Stress

As effective components of microbial biofertilizers, the resistance of functional strains to abiotic stress directly affects their root colonization capacity, thereby affecting their efficacy as biofertilizers [46,47]. Researchers have found that aluminum-tolerant microbes, isolated from the acidic soils of rice rhizospheres, are more readily adapted to rice ecosystems due to their stable colonization capabilities and minimal negative impact on the environment [26]. This strategy of reintroducing native microbes, as opposed to introducing exotic species, can more effectively promote the establishment and persistence of functional microorganisms within the plant–soil-microbe system. To mitigate the inhibition of the growth and colonization of KSR under alkali stress, we isolated alkali-tolerant KSR from local soil. We observed that KSR exhibited significant alkaline tolerance in environments at pH 7 to 9, suggesting their ability to thrive across a wide pH spectrum. Notably, these bacteria demonstrated optimal growth at pH 8 to 9. Furthermore, the mechanism by which phosphate-solubilizing bacteria release phosphate is primarily through acidolysis and enzymatic hydrolysis [48]. Research indicates that the production of organic acids and the activity of these enzymes are influenced by factors including pH, temperature, and the type of carbon source. Therefore, it is crucial for farmers to consider the soil’s nutrient content and physicochemical properties when selecting phosphate-solubilizing bacterial fertilizers [49]. Similarly, it is essential to verify whether the potassium-solubilizing function of KSR is significantly inhibited under alkaline stress. Our results indicated that KSR strains maintained high potassium-solubilizing activity at pH 7 to 9, with some strains displaying their peak performance at pH 8. Strain A10 produced various organic acids at pH 7 to 10, with the peak concentration of acetic acid observed at pH 10. These findings not only confirm the adaptability of KSR strains to alkaline conditions but also highlight their potential as microbial fertilizers for enhancing potassium availability in alkaline soils.

Further analysis revealed significant associations between KSR proliferation, pH dynamics, and potassium liberation. In the early phase of its cultivation, *Pseudomonas aeruginosa* A10 grew slowly, with minimal fluctuations in both pH and soluble potassium concentration. Subsequently, fueled by the released potassium, isolate A10 proliferated rapidly and secreted acidic substances that lowered the environmental pH, hastening the dissolution of mineral potassium. In the later stages of its cultivation, KSR A10’s growth stabilized or declined, while the secretion of organic acids stabilized or decreased. Following this, processes such as cellular decomposition, regeneration, and potassium liberation occurred simultaneously, resulting in a gradual increase in the soluble potassium concentration.

### 4.4. Plant Growth of Cotton in the Presence of KSR

Potassium deficiency inhibits root growth in crops, resulting in significant declines in plant growth and yield [50]. Reviews have demonstrated that KSR enhance plant growth by solubilizing potassium [17]. For example, the KSR isolate *Mortierella* 2K4, obtained from the rhizosphere soil of poplar, has been shown to enhance maize seedlings’ growth, increasing both the fresh and dry biomass of their shoots and roots [51]. Similarly, the KSR isolate *Klebsiella* XF11, found in tobacco rhizosphere soil, promotes potassium uptake in tobacco, increasing plant biomass [52]. The root–shoot ratio is an important indicator reflecting the resource allocation strategies of plants under various growth conditions, providing insights into their growth, adaptation, and competitiveness [53]. This research investigated the impact of isolate A10 on cotton seedlings’ growth and root–shoot ratio. The results from pot experiments indicated that KSR significantly enhanced both the shoot and root growth of cotton seedlings while reducing the root–shoot ratio. This suggests that KSR alleviated nutritional stress in the cotton rhizosphere, leading to greater resource allocation to the shoots. These results underscore the promise of *Pseudomonas aeruginosa* A10 as a biofertilizer.

However, as these results were obtained under greenhouse conditions, further validation is needed to confirm its efficacy in natural environments. Additionally, a study [54] found that the virulence factor redox-active pyocyanin produced by *Pseudomonas aeruginosa* PAO1 can regulate root development without causing obvious toxic symptoms in the host plant. This finding suggests that the activity of redox-active pyocyanin in plants significantly differs from that in animal cells. Moreover, *Pseudomonas aeruginosa* M18 exhibits biocontrol activity, protecting plants from pathogen infection. Notably, the genome of *Pseudomonas aeruginosa* M18 lacks all genomic islands and prophages related to mammalian pathogenicity reported in other studies [55]. This indicates that *Pseudomonas aeruginosa* strains from different environmental niches show significant differences in their genome expression profiles and virulence activities. Therefore, it is crucial to completely sequence *Pseudomonas aeruginosa* A10 isolated from the cotton rhizosphere and analyze it as a model strain to evaluate its pathogenicity and cytotoxicity, ensuring the safety of its development and application. When applying this bacterium to natural environments, we will implement rigorous risk assessment and management strategies to monitor and control its environmental spread. This includes studying its interactions with non-target plants and evaluating the ecological impact of its release. By addressing these safety concerns, we aim to develop microbial fertilizers that are both effective and environmentally safe.

## 5. Conclusions

The application of KSR to soil–plant systems represents an effective nature-based solution for alleviating environmental stress and enhancing crop productivity. We screened potential alkali-tolerant KSR strains from the rhizosphere soil of cotton in Xinjiang. Our experimental results demonstrated that *Pseudomonas aeruginosa* A10 exhibited a favorable potassium solubilization capability (21.78 ppm). Moreover, it thrived and maintained its solubilization ability in weakly alkaline environments (pH 7–9). This bacterium primarily promotes potassium solubilization by secreting organic acids such as lactic, citric, acetic, and succinic acid. Additionally, KSR A10 secretes these organic acids at pH 7 to 9, maintaining its potassium-dissolving ability effectively. Greenhouse pot experiments showed that inoculating cotton plants with strain A10 increases their biomass and reduces their root–shoot ratio. These findings underscore the potential of KSR to improve potassium availability in alkaline cotton fields and enhance our understanding of potassium nutrient cycling in soil. Further research should explore KSR’s potential to enhance other soil nutrients and evaluate its field applications.

## Figures and Tables

**Table 1:** Soil properties of the rhizosphere of cotton.

Total Potassium (g·kg^−1^)	Available Potassium (mg·kg^−1^)	Total Phosphorus (g·kg^−1^)	Total Nitrogen (mg·kg^−1^)	Soil Moisture (g·g^−1^)	pH
15.61 ± 1.21	148.11 ± 7.13	1.19 ± 0.07	0.59 ± 0.13	0.13 ± 0.10	8.28 ± 0.11

Each value is the average of a minimum of three replicates.

**Table 2:** Soluble potassium content (ppm) in broth with KSR strains under various alkaline stress conditions.

Strain	pH = 7	pH = 8	pH = 9	pH = 10
A1	8.25 ± 0.94 aCDE	7.49 ± 0.50 abCD	7.07 ± 0.42 bC	3.11 ± 0.06 cB
A6	10.30 ± 0.74 aA	9.49 ± 1.24 abAB	8.71 ± 0.68 bB	3.76 ± 0.07 cA
A9	6.50 ± 1.40 aFGH	6.12 ± 0.55 aEF	5.13 ± 0.11 aF	2.35 ± 0.09 bEFG
A10	10.70 ± 0.91 aA	10.41 ± 0.98 aA	9.63 ± 0.18 aA	4.24 ± 0.12 bA
A12	5.80 ± 0.56 aGH	4.67 ± 0.40 bG	4.02 ± 0.13 bGH	2.75 ± 0.14 cBCDE
A15	9.45 ± 0.78 aABC	9.19 ± 0.90 aB	8.56 ± 0.35 aB	4.09 ± 0.08 bA
A16	7.50 ± 0.17 aDEFG	7.13 ± 0.31 aCDE	5.40 ± 0.35 bF	2.94 ± 0.10 cBCD
B4	6.85 ± 0.96 aEFGH	6.82 ± 0.39 aDE	6.08 ± 0.22 aE	2.21 ± 0.11 bFGH
B5	7.50 ± 1.10 aDEFG	6.37 ± 0.50 aEF	4.21 ± 0.12 bGH	2.61 ± 0.09 cCDEF
B7	5.85 ± 0.09 aGH	4.94 ± 0.14 bG	4.25 ± 0.07 cGH	2.13 ± 0.06 dFGHI
B9	5.20 ± 0.18 aI	5.03 ± 0.24 aG	4.31 ± 0.17 bGH	1.71 ± 0.10 cIJ
B11	7.85 ± 1.82 aDEF	8.10 ± 0.45 aC	7.24 ± 0.33 aC	2.36 ± 0.18 bEFG
B12	4.25 ± 0.78 aI	4.57 ± 0.29 aG	4.41 ± 0.17 aG	1.36 ± 0.13 bJ
C1	7.20 ± 1.04 aDEFG	5.37 ± 0.38 bFG	5.05 ± 0.18 bF	3.23 ± 0.11 cB
C2	8.57 ± 0.52 aBCD	7.72 ± 0.67 bCD	6.54 ± 0.18 cD	1.99 ± 0.11 dGHI
C4	6.70 ± 1.57 abEFGH	6.87 ± 0.54 aDE	5.22 ± 0.13 bF	2.55 ± 0.07 cDEF
C6	3.70 ± 0.99 aI	4.52 ± 0.16 aG	3.89 ± 0.09 aH	1.76 ± 0.05 bHIJ
C13	7.00 ± 0.66 aDEFG	7.15 ± 0.62 aCDE	7.02 ± 0.17 aC	3.09 ± 0.52 bBC
C14	6.55 ± 0.09 aEFGH	6.68 ± 0.47 aDE	6.57 ± 0.38 aD	2.30 ± 0.34 bEFG
C16	9.78 ± 0.78 aABC	9.25 ± 0.33 abB	8.47 ± 0.26 bF	3.21 ± 0.56 cB
h4	10.05 ± 0.26 aABC	10.09 ± 0.85 aAB	9.74 ± 0.14 aA	4.01 ± 0.79 bA
s1	7.15 ± 0.95 aDEFG	6.33 ± 0.30 abEF	5.40 ± 0.07 bF	3.82 ± 0.32 cA
LJ	6.45 ± 0.26 aFGH	6.81 ± 0.57 aDE	5.14 ± 0.10 bF	2.77 ± 0.23 cBCDE

Each data point represents the mean of at least three replicates. Different lowercase letters indicate significant differences among different alkali treatments under the same inoculation condition (*p* < 0.05), and different uppercase letters indicate significant differences among different inoculation conditions under the same alkali treatment (*p* < 0.05).

**Table 3:** Effect of KSR A10 on organic acid secretion under varying alkalinity.

Organic Acid	Oxalic Acid (μg/mL)	Tartaric Acid (μg/mL)	Pyruvic Acid (μg/mL)	Lactic Acid (μg/mL)	Acetic Acid (μg/mL)	Citric Acid (μg/mL)	Succinic Acid (μg/mL)
pH = 7	2.49 ± 0.23 a	2.28 ± 0.12 b	9.71 ± 0.68 a	147.09 ± 10.32 a	55.12 ± 3.44 b	49.65 ± 1.68 d	35.12 ± 4.33 b
pH = 8	2.23 ± 0.19 a	2.86 ± 0.20 a	8.15 ± 0.83 b	92.15 ± 5.56 b	21.73 ± 1.80 c	107.36 ± 5.57 a	57.00 ± 5.49 a
pH = 9	2.56 ± 0.13 a	2.23 ± 0.20 b	6.91 ± 0.60 bc	87.65 ± 7.27 b	64.33 ± 5.29 b	79.26 ± 1.65 b	20.91 ± 2.28 c
pH = 10	2.50 ± 0.26 a	0.75 ± 0.12 c	6.62 ± 0.67 c	35.05 ± 3.47 c	109.22 ± 7.94 a	67.29 ± 4.21 c	15.57 ± 1.51 c

Each data point represents the mean of at least three replicates. Different lowercase letters in the same column indicate significant differences in organic acid content among different alkaline treatments (*p* < 0.05).

**Table 4:** Effects of KSR A10 on cotton growth.

Treatment	Root		Aboveground		Root–Shoot Ratio
	Total Length (cm)	Dry Weight (g)	Plant Height (cm)	Dry Weight (g)	
Control	377.04 ± 42.51 d	0.60 ± 0.05 c	10.56 ± 0.71 d	1.15 ± 0.40 d	0.52
K	437.26 ± 48.50 c	0.64 ± 0.04 b	11.73 ± 0.50 c	1.25 ± 0.12 c	0.51
A10	473.80 ± 18.07 b	0.67 ± 0.04 b	12.95 ± 0.71 b	1.35 ± 0.09 b	0.50
K + A10	533.05 ± 29.21 a	0.74 ± 0.03 a	14.22 ± 0.57 a	1.68 ± 0.08 a	0.44

Each value is the average of a minimum of fifteen replicates. Different lowercase letters in the same column indicate inoculation treatments with significant differences (*p* < 0.05).

## Data Availability

Information about *Pseudomonas aeruginosa* A10 is available in the National Center for Biotechnology Information (NCBI) database, link: https://www.ncbi.nlm.nih.gov/nuccore/PP516541.1/, accessed on 27 March 2024. The raw data supporting the conclusions of this article will be made available by the authors upon request.

## Supplementary Materials

The following supporting information can be downloaded at https://www.mdpi.com/article/10.3390/microorganisms12071474/s1, Figure S1. Effect of KSR on morphological attributes of cotton seeding under alkali stress conditions; Table S1. Morphological characteristics of different strains.
