# Supplementary material for: Isolation and Characterization of Potassium-Solubilizing Rhizobacteria (KSR) Promoting Cotton Growth in Saline–Sodic Regions
Source: Microorganisms. 2024 Jul 19;12(7):1474. doi: 10.3390/microorganisms12071474 (PMC11279176; doi:10.3390/microorganisms12071474)

Table S1. Morphological characteristics of different strains.

Strains	Form	Edge	Color	Opacity	Texture	Adhesion	Elevation	Surface
**A1**	Circular	Serrated	yellowish	Transparent	Liquid	Viscous	Flat	Smooth, Glossy
**A6**	Circular	Serrated	yellowy	Transluscent	Liquid	Non-adhesive	Flat	Smooth, Matte
**A9**	Circular	Regular	yellowish	Transluscent	Viscid	Adhesive	Flat	Smooth, Matte
**A10**	Circular	Undulate	yellowy	Transparent	Liquid	Adhesive	Flat	Smooth, Glossy
**A12**	Circular	Serrated	yellowy	Transluscent	Viscid	Adhesive	Flat	Smooth, Matte
**A15**	Circular	Regular	yellowish	Transluscent	Liquid	Adhesive	Flat	Smooth, Matte
**A16**	Irregular	Regular	yellowy	Transparent	Liquid	Non-adhesive	Flat	Smooth, Glossy
**B4**	Irregular	Undulate	brown	Opaque	Viscid	Adhesive	Convex	Rough, Matte
**B5**	Circular	Regular	brown	Opaque	Viscid	Adhesive	Flat	Smooth, Matte
**B7**	Circular	Undulate	yellowy	Transluscent	Viscid	Adhesive	Flat	Smooth, Matte
**B9**	Circular	Regular	celadon	Opaque	Liquid	Non-adhesive	Flat	Smooth, Matte
**B11**	Circular	Undulate	taupe	Transluscent	Liquid	Non-adhesive	Flat	Smooth, Matte
**B12**	Circular	Undulate	brown	Transluscent	Viscid	Adhesive	Flat	Smooth, Matte
**C1**	Circular	Serrated	brown	Opaque	Viscid	Adhesive	Flat	Rough, Matte
**C2**	Circular	Serrated	brown	Transparent	Liquid	Adhesive	Convex	Rough, Matte
**C4**	Circular	Undulate	brown	Transluscent	Viscid	Adhesive	Flat	Smooth, Matte
**C6**	Circular	Regular	yellowy	Transluscent	Viscid	Adhesive	Flat	Smooth, Matte
**C13**	Circular	Regular	brown	Opaque	Viscid	Adhesive	Flat	Rough, Matte
**C14**	Circular	Regular	yellowish	Transluscent	Liquid	Adhesive	Flat	Smooth, Matte
**C16**	Circular	Serrated	yellowy	Transluscent	Liquid	Non-adhesive	Flat	Smooth, Matte
**h4**	Irregular	Undulate	yellowy	Transluscent	Liquid	Non-adhesive	Flat	Rough, Matte
**s1**	Irregular	Undulate	brown	Opaque	Viscid	Non-adhesive	Flat	Rough, Matte
**LJ**	Circular	Undulate	yellowish	Transparent	Liquid	Adhesive	Flat	Smooth, Matte

Figure S1. Effect of KSR on morphological attributes of cotton seeding under alkali stress condition.

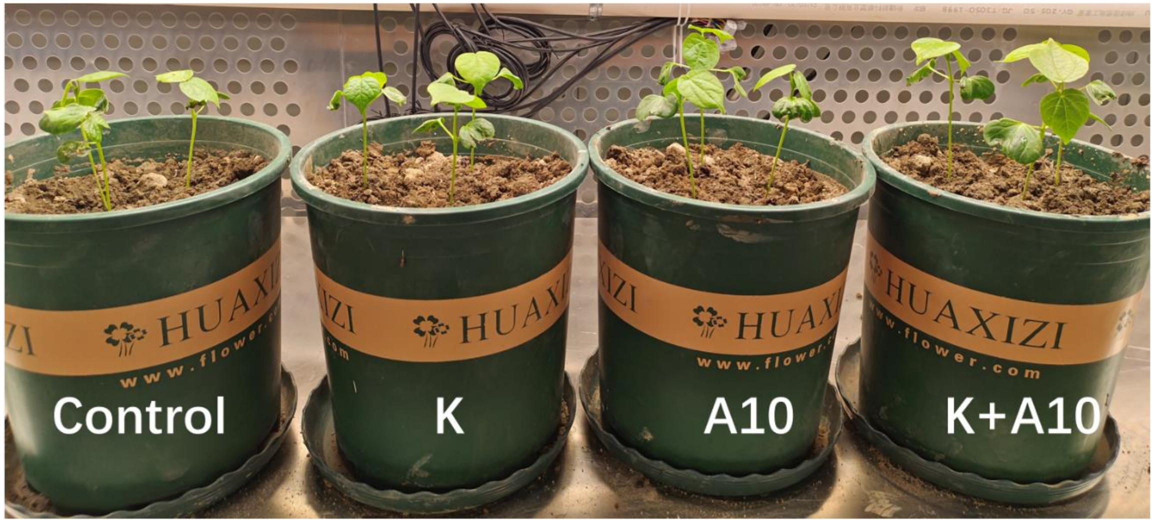

Supplement: Supplementary file 1 [file microorganisms-12-01474-s001.zip › microorganisms-3089921-SI.pdf]
